# How are Autistic People Involved in the Design of Extended Reality Technologies? A Systematic Literature Review

**DOI:** 10.1007/s10803-023-06130-3

**Published:** 2023-09-16

**Authors:** Nigel Newbutt, Noah Glaser, Marc Sonley Francois, Matthew Schmidt, Sue Cobb

**Affiliations:** 1 School of Teaching and Learning, Institute of Advanced Learning Technologies, University of Florida, Gainesville, FL USA; 2 School of Information Science & Learning Technologies, University of Missouri, Columbia, MO USA; 3 Faculty of Engineering, University of Nottingham, Nottingham, UK

**Keywords:** Extended reality, Immersive technology, Autistic, Virtual reality, Augmented reality, Systematic review, Co-design

## Abstract

The primary aim of this systematic review is to investigate the inclusion of autistic individuals in the design process of immersive technologies. This study follows the preferred reporting items for systematic reviews and meta-analyses standards for systematic literature reviews. To ensure the research questions and subsequent stages of the review incorporate pertinent parameters, the problem, interest, context framework has also been employed. Findings highlight that, while early proponents of immersive technology emphasized the importance of user involvement in design of new technology, immaturity of the technology often limited the implementation of direct user input to the design process. Nonetheless, analysis of the literature published between 2002–2022 identified 20 studies in which substantial influence of autistic individuals and stakeholders was found in the design process of immersive technologies. The roles of autistic individuals varied from active co-designers and co-creators to essential contributors in refining prototypes and providing critical feedback, ensuring the final products align with their needs and preferences. Results underscore the need to align research and design of immersive technologies more closely with the priorities and preferences of autistic individuals. Further is needed regarding actively involving autistic individuals in the design and implementation of immersive technology applications. On this basis, we maintain that more inclusive and effective deployment of immersive technologies is needed in order to ensure that resultant technologies are fit for purpose and address the actual needs of the autistic community.

**Supplementary Information:**

The online version contains supplementary material available at 10.1007/s10803-023-06130-3.

## Introduction

Extended reality technologies have been recognized for their unique potential in supporting training and learning for autistic individuals^1^ (Parsons & Mitchell, 2002). Since mid-1990s, these technologies have been investigated in research settings, with early studies focusing primarily on autism user tolerance and acceptance of extended reality technology and evaluation of effectiveness for supporting social skills, emotional and behavioral regulation, executive functioning, and daily living skills (Mesa-Gresa et al., 2018; Lorenzo et al., 2019). As digital and extended reality technologies have advanced, the concept of ‘Extended Reality’ (XR) has been used as an umbrella term for immersive learning technologies, including virtual reality (VR), augmented reality (AR), virtual worlds (VW), mixed reality, and “all realities on the mixed reality spectrum” (Lion-Bailey et al., 2023, p.123). Virtual Reality (VR) comprises computer-simulated representation of a world (i.e., a virtual environment) with specific spatial and physical characteristics. Sensory display and interaction peripherals provide a fully-immersive experience, for example, by wearing a head-mounted display such that the real-world is no longer visible, or using non-immersive displays such as desktop-based computer monitors or projectors. Augmented reality refers to digitized overlays on a real-world environment. These overlays can typically be seen either though a handheld platform (e.g., smartphone) or hands-free headset (e.g., AR glasses). Mixed reality, which also includes digitized overlays on the real world, allows users to manipulate or use the environment to show or control parts of the digitized content (Speicher et al., 2019).

For over 30 years, research in autism and the XR field has steadily progressed, and interest has surged since the introduction of commercially available, off-the-shelf extended reality technologies like the Oculus Rift and low-tech alternatives, such as Google Cardboard (Parsons et al., 2017). While immersive experiences offered by these technologies are thought to hold promise for autistic users (Parsons, 2016), the empirical evidence supporting their effectiveness still faces significant challenges (Glaser & Schmidt, 2022). The current research on the use of extended reality technologies has been characterized as fragmented and unsystematic (Parsons, 2016). Research employing these technologies and their definitions vary greatly (Glaser & Schmidt, 2022), with a glaring lack of theoretical foundation (Schmidt & Glaser, 2021a, b). Moreover, the assertion that skills acquired in VR will transfer to real-life contexts remains inadequately substantiated (Schmidt et al., 2023). Designing extended reality technologies and conducting well-controlled research studies has proven to be an intricate task. Some researchers have even described it as a “wicked problem” (Schmidt, 2014; Schmidt & Glaser, 2021a, b), highlighting the complex nature of integrating immersion with research and practice for the benefit of autistic individuals. The purpose of this study is to understand that ways that autistic people have been involved in research about them in the field of XR (as defined above). To frame this, the remainder of this literature view will outline the need for greater inclusion of autistic groups in research that focuses on/about them (Pellicano et al., 2014), while also arguing that involving end-users (autistic people) in the design process has often been a promise and ambition in this field rather than a reality. We end with an overview of why and how XR technologies are seen as a feasible, and sometimes beneficial, technology for autistic groups and that without greater inclusion the field is missing opportunities for community engagement while neglecting the priorities and preferences of the autistic community. This results in XR technologies often being designed without any direct input from autistic people.

In the general and mainstream autism literature, a mismatch has been identified between the kinds of research that is funded and the kinds of research that is valued by autistic groups (Pellicano et al., 2014). For example, substantial amounts of funding tends to support research in areas such as genetics, neural systems, and developmental and behavioral interventions (Den Houting & Pellicano, 2019; Frazier et al., 2018). However, research suggests that autistic people place greater value on research that focuses on adult transition, lifespan issues, and health and well-being (Harris et al., 2021). This mismatch extends to research on extended reality technologies involving autistic groups, which has predominantly focused on social skills and emotional skills, as opposed to skills related to daily living, phobias, and physical activities (Mesa-Gresa et al., 2018). Interestingly, in a meta analysis by Karami and colleagues (2020), researchers found that VR systems focusing on social and communication skills, emotion regulation and recognition, and executive functioning were less effective than those focusing on daily living skills (Karami et al., 2020), suggesting a further mismatch between the areas in which extended reality technologies currently are used and the areas in which they are most likely to prove efficacious.

Early proponents of extended reality technology for social skills training emphasized the importance of involving end-users in the design process (Parsons et al., 2000; Parsons & Mitchell, 2002; Kerr et al., 2002; Rutten et al., 2003). However, the limited advancements of the technologies in these early studies resulted in a lack of long-term implementation (Newbutt, 2013). Consequently, only minimal understanding was gained about the advantages of directly involving autistic users in designing extended reality technology applications. In more recent research, efforts have been made to prioritize the input of autistic individuals and their stakeholders by documenting their involvement in the design process. For instance, Newbutt and Bradley (2022) describe their collaborative work with a school for autistic students, focusing on research involving VR. They employ ethical and participatory approaches to ensure the inclusion of autistic voices, allowing these individuals to shape and direct the research that involves them. Similarly, Schmidt et al. (2021) summarize their procedures to emphasize safe working practices when using VR head-mounted displays (HMDs) with autistic individuals. In doing so, they advocate for the inclusion of autistic individuals and stakeholders (e.g., teachers, parents) in guiding research and establishing protocols. Both of these studies arrived at their conclusions by actively involving autistic participants in their research, providing them with meaningful opportunities to contribute to the development and implementation of extended reality technologies.

Since the early work of Parsons et al. (2000); Parsons & Mitchell (2002); Kerr et al. (2002); Rutten et al. (2003), the inclusion of autistic users in designing extended reality technologies has seen limited progress. However, research employing co-design approaches for general technology development (i.e., non-immersive technologies) with autistic individuals has highlighted several benefits for both researchers and autistic co-designers (Politis et al., 2017). Notable examples of inclusive design can be found in the broader autism research. For instance, Benton et al. (2014) discuss the successful implementation of a structured method for inclusive design with autistic children called IDEAS (Interface Design Experience for the Autistic Spectrum). Their results indicate that autistic children, with support, actively participated in the design process (Benton, 2014), and the experience was largely positive. Moreover, the participatory approach benefited both autistic and neurotypical children, albeit not uniformly (Benton & Johnson, 2014). Additionally, Zhu et al. (2019) explored how autistic adolescents could collaborate as co-designers in an iterative software design process. They found that the autistic co-designers enjoyed the process and engaged in meaningful design discussions. The researchers also gained insights into the local autistic community and built positive relationships before the co-design workshops. Similarly, Bossavit and Parsons (2018) carried out a pilot study to explore and analyze an academic-based educational game co-designed with and for autistic youth. The game focused on enhancing their knowledge of Geography. The findings of the study revealed that the participants had a positive experience with the game, reporting increased enjoyment, motivation, and social engagement. Furthermore, their knowledge of Geography content also increased as a result of the educational game. In conclusion, the researchers suggest that co-design can better accommodate and support children's diverse interests and preferences (Bossavit and Parsons, 2018).

The importance and value of including autistic people may seem obvious, but it is an issue that has, and continues, to elude the field of designing XR experiences for autistic participants. Reports of autistic input to research studies, at any stage of the process, are rare (Monahan et al.; 2023). We recognize that there is not always space in formal research outputs (i.e. journal articles and conference proceedings) to report the process of autistic inclusion and co-design. Strict word limits in journals and book chapters can limit this; however this should not limit the capacity of researchers to include the priorities, ambitions, and input for autistic stakeholders in their research. An example of this is in work examining the views on researcher-community engagement in autism research by Pellicano and colleagues (2014), who found that “researchers perceive themselves to be engaged with the autism community but that community members, most notably autistic people and their families, did not share this view” (p. 1). Within technology, and specifically, XR research, limitations in community engagement stand to neglect the priorities and preferences of the autistic community; meaning technology is often being designed without any direct input from autistic people, either from the outset, in defining needs, or the design and development phases. This could be one reason that, to date, very few research projects extend beyond controlled laboratory settings to have actual in-situ impact on the lives of autistic people. Therefore, we present the current study in the context of locating where co-production and co-design has taken place in the field of designing XR experiences, seeking to describe the characteristics, the nature of inclusion, and the reported outcomes, recommendations, and implications of co-design with autistic people.

### Aims of this Review

Previous reviews have shown that XR technologies are feasible and sometimes beneficial for teaching autistic children a range of skills (e.g. Berenguer et al., 2020; Mesa-Gresa et al., 2018; Lian & Sunar, 2021; Savickaite et al., 2022). For example, Mesa-Gresa and colleagues (2018) conducted a systematic review of the literature incorporating both clinical and technical databases on the effectiveness of VR-based systems for autistic individuals. Their review of 31 articles found moderate evidence supporting effectiveness of VR in training. Similarly, Berenguer and colleagues (2020) conducted a systematic review that investigated the impact of AR on the social, cognitive, and behavioral domains in children and autistic adolescents. Their analysis of 20 selected articles from an initial pool of 387 records suggested promising results for AR-based treatments in improving health and wellbeing in young autistic people, setting a path for future exploration in this area. Another systematic review analyzed 82 articles resulting in 49 records, found inconsistencies in how VR is defined and designed, which could significantly affect the potential benefits of these technologies and the possibilities for user interaction (Glaser & Schmidt, 2022).

To our knowledge, no systematic review yet has investigated how XR technologies are designed through participatory or co-design approaches with autistic groups. Without the specific and vital input of autistic people, the risk exists that XR design and uptake will remain limited and ethical implications of not including autistic people in this research agenda will remain unexplored (Poulsen et al., 2022). Furthermore, we were unable to locate any reviews that place a focus on, or examine, ways in which research has involved or included the role of autistic users in the design process of XR. The importance of inclusion is highlighted in a systematic review from the related research field of serious games, in which Tsikinas and Xinogalos (2019) reviewed 54 articles published between 2005–2018. The researchers note that only 7 included details of the design methodology applied, concluding that “Even though the design methodology is presented in a limited number of studies, it is observed that involving end users or professionals in the field of special education is preferred, either by using the participatory design, user- or learner-centered approaches.” (p.70).

Therefore, in this paper we examine the extent that autistic people have been involved or included in the design of XR systems for autistic individuals. This is operationalized through the following three research questions:

1. What are the characteristics of published projects that include autistic people in extended reality technology-based research?
2. What are reported outcomes, recommendations for, and implications of participatory and co-design with autistic people for research and practice?
3. What is the nature of inclusion of autistic people in the corpus of included articles?

## Methods

The current systematic literature review was carried out, following the Preferred Reporting Items for Systematic Reviews and Meta-Analyses (PRISMA) standards (Moher et al., 2009). Using the PRISMA checklist establishes trustworthiness by allowing transparency and replicability of the results in subsequent studies (Page et al., 2021). In addition, to guarantee that the research questions and subsequent stages of our review incorporate pertinent parameters, we employed the Problem, Interest, Context (PICo) framework (Stern et al., 2014).

- Population (P): autistic individuals and stakeholders (RQ1)
- Interest (I): promoting inclusion and engagement of autistic individuals in the research development and implementation process (RQ2)
- Context (Co): research initiatives that utilize XR technology to provide supports for autistic individuals (RQ3)

The subsequent subsections provide a summary of the protocol registration, eligibility criteria, information sources, search strategy, and selection process.

### Protocol Registration

The protocol for this review was registered as “Is inclusion and involvement of autistic people actually taking place in extended reality technology development and application of research? A systematic literature review” in the Open Science Framework (OSF) at osf.io/hnzur on September 13, 2022. Appendix A (in supplementary materials) list the database and search terms used.

### Eligibility Criteria

To identify high-quality articles that address our research questions, we crafted a set of criteria for inclusion and exclusion of the articles (see Appendix B). These criteria are as follows: (1) only peer-reviewed articles published in academic journals, accessible through the selected databases, and in English were included. Editorials, book chapters, conference abstracts, conference proceedings, retracted publications, and studies not published in English were excluded to ensure the quality of the articles and the value of their outcomes; (2) we limited our search to the studies published between 2002 and 2022 to capture 20 years of published research that encapsulates contemporary developments in the XR field; (3) this systematic literature review aims to identify original articles and not previous reviews, meta-analyses, or systematic literature reviews; (4) to be included, the research project had to either apply a participatory design approach or be co-designed with autistic individuals or stakeholders and use extended reality technologies; (5) studies involving autistic individuals or stakeholders solely in evaluating their final products were excluded; (6) studies that included a range of developmental or intellectual conditions in addition to autism were also excluded. (7) studies that did not provide sufficient details of empirical research design and data analysis were not included; and finally, (8) conceptual or descriptive studies (e.g., discussing the potential benefits of using extended reality technologies for the autistic community) were excluded from this review.

### Information Sources

We used a two-stage search strategy to mitigate the risk of missing relevant articles. In stage one, we performed an advanced search in the following four databases: Web of Science, Pubmed, Scopus and EBSCO (Academic Search Premier). In stage two, we manually searched the reference lists of all identified relevant articles using ancestral searching (reviewing references in the literature of our included articles), which reduced the risk of missing relevant studies (Wohlin, 2014). Additional articles found in stage two that appeared to be eligible for consideration were assessed using the same study selection criteria used for the main search selection. The study utilized electronic databases pertinent to education, IT, and medical research with broad scientific literature coverage. Web of Science was selected for its multidisciplinary, independent nature, and its alignment with research queries, offering vast citation data. PubMed, a free database with over 35 million biomedical literature citations, aims to enhance health outcomes globally. Scopus, Elsevier's multidisciplinary citation database, covers a range of disciplines including arts, medicine, science, social sciences, and technology. Lastly, Academic Search Premier provides access to nearly 3000 journals and magazines, including 1000 active, full-text, peer-reviewed journals with no embargo, covering diverse topics from allied health to humanities.

### Search

This study involved electronic database searches using keywords determined by the researchers (see Appendix C) and ancestral searching. Three researchers first defined a list of terms focused on autism, immersive technologies, and co-design to build the initial search strings. A pilot search was conducted on all data sources to verify the relevance and effectiveness of the resulting search strings. Based on the results, an iterative approach was used to allow the authors to revise string terms, which helped avoid some unwanted outcomes being returned. Databases were searched between March 2022 and January 2023. Of note, the search terms used in all databases were fundamentally the same. However, minor modifications were applied for each database to meet their formatting requirements, as detailed in Supplementary Materials. (Appendix D).

### Search Results Reliability

To ensure the reliability of the search results, a subsequent search using the same search strategy, including queries, keywords, and filters, was conducted by one of the co-authors one week after the initial search across the databases. The primary objective of the subsequent search was to confirm the number of returned results. The results of the subsequent search demonstrated a perfect agreement with the initial search results.

### Study Selection

The search results were imported into Zotero, an open-source reference management software program, to manage the bibliographic data and generate an integrated file. In addition, the file was transferred to Covidence (Covidence systematic review software, 2023), an online software that automatically removes duplicates and facilitates the review and selection of articles according to the PRISMA approach. Using Covidence, the first and second authors independently screened each title and abstract based on the eligibility criteria. If the studies met the inclusion criteria, they were carried forward to the full-text review. Studies deemed out-of-scope based on the full-text review were excluded with a documented rationale. Any disagreements between the two reviewers were discussed and resolved through consensus at each stage. If inclusion of an article was uncertain after discussion, a third reviewer was consulted.

### Study Selection Inter-Rater Reliability

To evaluate the level of agreement between reviewers in the study selection process, we used Cohen's Kappa, a statistical measure of inter-rater reliability (Cohen, 1960). The Kappa score obtained was 89.99%. The literature indicates that this agreement coefficient demonstrates acceptable inter-rater reliability, as described by various terms such as "moderate" (McHugh, 2012), "strong" (Schober et al., 2018), or "high positive" (Hinkle et al., 2003) indicating a substantial degree of consistency and reliability in the raters' selection of studies.

### Data Collection Process

To thoroughly analyze the articles included in this review, two researchers jointly extracted meaningful data from each paper. To this end, a spreadsheet was created to ensure that all study information was stored in one place, making it easier to compare and analyze the data extracted during the synthesis process. Storing the data in a spreadsheet also allowed the research team to easily filter and analyze the data.

### Data Items

Data extracted from each manuscript comprised the following:

- *Article source* details about the article, such as its title, authors, publication date, and where it was published, disciplines, and type of study.
- *Target audience* autism diagnosis details and age range of autistic people.
- *Description of technologies* AR, VR, Mixed Reality or Virtual Worlds.
- *Study characteristics* the aim of the research, research methodology, and who the informants were.
- *Description of the design process* description of co-design, at what step/stage of the research were the autistic individuals involved, how did the design get informed/was adjusted by this process, challenges of co-design, what did the co-design approach lead to for autistic people, what were the outcomes of the process, implications for practice, and future work statement.

## Results

**Fig. 1:**
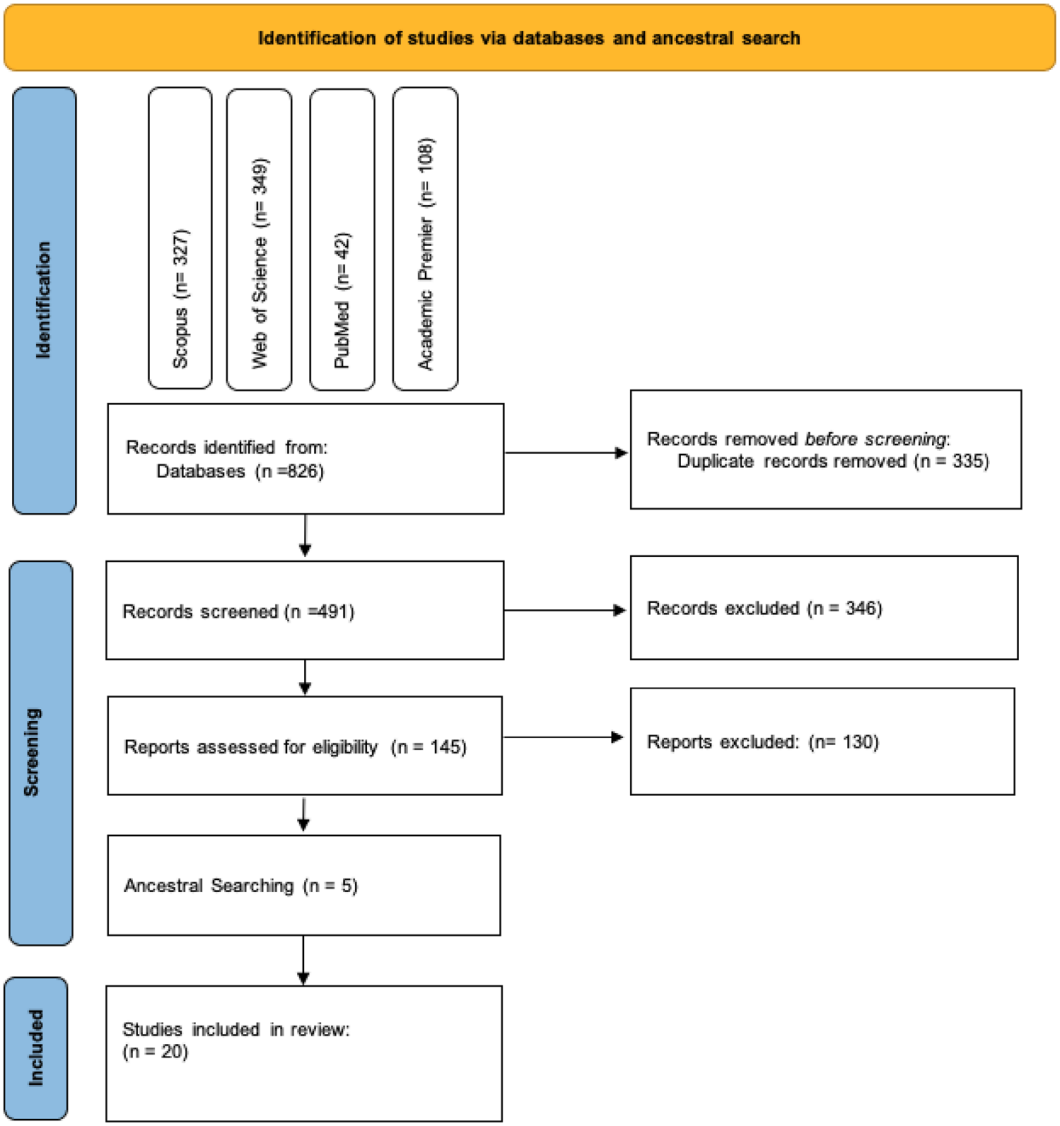
Flow chart of the literature identification and screening process

This section presents the key findings from the analysis of the selected articles with respect to the three research questions listed previously. Our initial search generated 826 records, plus additional records (n = 11) identified from ancestral searches. After eliminating duplicates (n = 335), 491 records were screened according to their title and abstract. Of these, 335 articles were removed due to their irrelevance to the research questions. As a next step, a full-text review was performed on the remaining 150 articles by three of the authors to determine their eligibility for inclusion in this review. Applying the inclusion criteria, 130 articles were eliminated. Reasons for exclusion of these 129 papers included: (1) the system was not evaluated by autistic individuals (n = 64), (2) autistic people and stakeholders were involved only in the evaluation of the final product (n = 58), and (3) insufficient detail was available to determine whether the research met the inclusion criteria (n = 8). As a result, a definitive collection of 20 articles was ultimately included. Figure. 1 illustrates the sequence and results of the search and selection process. Table 1 articulates the included studies and breakdown of their details.

**Table 1:** Detailed information of the included studies

Project title/description	Country	Participant details^a^	Technology used	Study location	System aim	Methodology
VR-JIT for transition-age youth with ASD (TAY-ASD); Smith et al., (2020)	USA	High-functioning ASD 16–21 years old (n = 24)	Hardware: VR-JIT is an internet-based tool designed to run on various devices such as computers, laptops, tablets, and headsets. Software: VR-JIT is a single-user computerized interview simulator with efficacy at enhancing interview skills and employment outcomes across three levels of difficulty (i.e., easy, medium, and hard).	School and home	Interviewing Skills	Mixed
AS Interactive; Cobb et al., (2002)	UK	Asperger’s syndrome/ High-functioning ASD 16–19 years old (n = na)	Hardware: Desktop-based VR with a mouse and a joystick. Software. Social Café offers both single-user virtual environments (SVEs) and collaborative virtual environments (CVEs). CVEs enable direct communication between participants within a shared virtual space, while SVEs involve user interaction with autonomous avatars.	Not described	Social skills	Qualitative
Rutten et al., (2003)	UK	Asperger’s syndrome/ High-functioning ASD Age not described	Hardware: Desktop-based VR with a mouse and a joystick. Software: Social Café offers both single-user virtual environments (SVEs) and collaborative virtual environments (CVEs). The SVEs were developed using the Superscape Virtual Reality Toolkit. The social café and formal meeting CVEs require the use of a network and run on both non commercializable software and software that entail acquiring a multi-user license.	School	Social skills	Qualitative
COSPATIAL; Millen et al., (2011)	UK	Details not described 16–17 years old (n = 5)	Hardware: Desktop-based VR with a mouse and keyboard. The scenario was projected via the interactive whiteboard in the classroom so that the whole group could see it. Software: Collaborative Virtual Environments (CVEs) that involve the utilization of Block Party, a shared problem-solving activity in which players work together to build blocks. Each player has different but interdependent objectives to achieve.	School	Social Skills	Qualitative
Museum accessibility; Giaconi et al., (2021)	Italy	High-functioning ASD Age not described (n = 5)	Hardware: Ricoh Theta SC2 spherical camera and a smartphone equipped with Android 6.0 OS. Software: The Ricoh Theta S apps, Ricoh Theta Converter Pro, Retouch3, and Marzipano Tool4. Marzipano Tool4 were utilized to create the initial sequence of spherical photos and organize them for the virtual tour.	Museum	Promoting cultural access	Qualitative
VR based behavioural learning; Ramachandiran et al., (2015)	Malaysia	Details not described Age not described (n = 41)	Hardware: Not described Software: Toilet virtual environment	Autism center	Behavioral skills	Mixed
Virtuoso; Schmidt & Glaser, (2021a, b)	USA	Mild and moderate ASD 22–34 years old (n = 5)	Hardware: Virtuoso-VR is compatible with HMD such as the Oculus Rift and can also be deployed as a desktop-based system using a wide range of input configurations (e.g., USB controllers) Virtuoso-SVVR is compatible with HMD that utilize smartphones such as the Google Cardboard and Google Daydream (now defunct) Software: Virtuoso-VR is a multi-user virtual environment developed in High Fidelity Virtuoso-SVVR is a single-user mobile application developed in Unity	University	Public transportation training	Multi
VL4ASD; Politis et al., (2020)	USA	Mild/moderate ASD > 20, < 30 years old (n = 3)	Hardware: Desktop-based VR Software: Virtual world developed using the Unity platform, whose main advantage is its high degree of customization that offers the opportunity to adapt training to better address individual needs.	Not described Not described	Communication skills	Quantitative
Politis et al., (2019)	Ireland	Mild/moderate ASD < 26 (n = 6), > 26 (n = 8)	Hardware: Desktop-based VR Software: Virtual world developed in the Unity platform, whose main advantage is its high degree of customization that offers the opportunity to adapt training to better address individual needs.	Home	Communication skills	Qualitative
MOBIS AR; Escobedo et al., (2014)	USA	Low-functioning ASD 3–8 years old (n = 12)	Hardware: Mobis architecture includes four main subsystems: the tag manager running on a PC, the Ambient Notification System (ANS) running on an Android smartphone, the therapy manager running on an Android tablet, and the augmented object embedded in the physical object. Software: Mobis is a mobile augmented reality application that lets teachers superimpose digital content on top of physical objects.	School	Improving attention	Mixed
AREmotion; Miningrum et al., (2021)	Indonesia	Details not described Age not described	Hardware: The AREmotion application was used on a smartphone with the following specifications: 2 GB RAM, 16 GB internal memory, a 16 MP camera for marker detection, and an Android 6.0 (Marshmallow) operating system. Software: AREmotion is a mobile application developed using Unity and Vuforia. The application combines an adapted book with augmented reality technology and is specifically designed to run on Android smartphones. The three menus of the application are video, ARAnimasi, and ARBook.	Not described	Emotional recognition	Qualitative
DIVRS for communication; Halabi et al., (2017)	Qatar	High-functioning ASD 4–6 years (n = 3)	Hardware: The participants in this study experienced VR via different installations: (1) a four walls projection room computer augmented virtual environment (CAVE) that provides a high level of immersion and interactivity (User wears polarized glasses with two markers on the rim of the glasses to enable tracking), (2) head mounted display (HMD- Oculus rift), and (3) non-VR normal desktop screen (20-inch LCD display). Software: The virtual environment and avatar were developed using Vizard from WorldViz. However, customized avatars and virtual scenes to create specific social communication environments were designed and generated using Autodesk’s Character Generator and rigged and animated using Autodesk’s 3Ds Max and Motion Builder software.	School	Communication skills	Quantitative
ARCB; Lee, (2019)	Taiwan	Moderate ASD 9–12 years old (n = 3)	Hardware: Participants viewed 3D animations of the ARCB materials on a tablet computer. Software: This system provides AR combined with a coloring book and presents corresponding specific elements in an AR 3D animation with dialogue using Quiver 3D AR coloring apps (http://www.quivervision.com/).	School	Social & communication skills	Quantitative
VR social stories; Ghanouni et al., (2019)	Canada	High-functioning ASD 15 years old (n = 1)	Hardware: Not Applicable Software: Not Applicable: This study developed a set of validated socio-emotional stories for autistic students, based on feedback from stakeholders. These stories have been designed to be used as content for a virtual reality program. However, the program has not yet been tested in a VR environment.	Fully Online	Social & emotional skills	Qualitative
VRCBT for social anxiety; Adams et al., (2022)	UK	Details not described 13 and 18 years (n = 5)	Hardware: Not Applicable: The HMDs were simply used to demonstrate to stakeholders (consultees) how they will be used in therapy. Software: Not Applicable: The study objective was to co-design the case series, treatment, and VR design protocols needed for testing their future system with autistic individuals and stakeholders. However, the VR design itself has not yet undergone testing.	Not described	Reducing social anxiety	Qualitative
CIRVR; Adiani et al., (2022)	USA	Details not described > 16 (n = 9)^b^	Hardware: Desktop-based VR with standard mouse, keyboard controls and a pair of headphones with a microphone. ***Note***: the authors designed two versions of CIRVR; one with a HMD and one without. The version of CIRVR reported in this article is the one without the HMD in order to capture facial image data. Software: CIRVR is a single user VR-based job interview training platform created using the Unity 3D Game Engine (version 2019) with a combination of purchased and custom-built elements.	Laboratory	Job interviewing skills	Mixed
VR for Affective Expression and Social Reciprocity; Ip et al., (2022)	China	Mild and High-functioning ASD 6–12 years old (n = 176)	Hardware: Oculus Rift headsets with Touch controllers were used. HMDs and controllers used support six-degree-of-freedom (6-DoF) tracking, meaning they could accurately track the translations and rotations of the user's head and hands in the VR space in real-time. Software: The virtual scenes and agents, along with their animations, were created using Blender. All required materials were imported into Unity, which was responsible for handling optimization techniques like baked lightmaps and reflection maps. The final VR scenarios were compiled into executable files using Oculus software.	School	Social and emotional skills	Quantitative
AR hand gesture and voice interaction; Amara et al., (2022)	Algeria	Details not described 2–12 years old (n = 18)	Hardware: The system includes a PC, an RGB camera, a Kinect device, and AR markers. The Kinect device is equipped with two cameras, an infrared camera and an RGB camera. Software: The AR System was developed in Unity3D using C# scripts. Two methods were employed in its creation. The first method utilized the ARToolKit plugin for Unity3D, while the second method involved embedding a virtual object within the Kinect video stream.	Autism center	Improving attention	Multi
Requesting help AR; Wang et al., (2022)	Taiwan	Low-functioning ASD 6–8 years old (n = 3)	Hardware: Mobile phone AR/ Scenes were displayed on a 20-inch monitor at a resolution of 1024 × 768 PPI. Software: Initially, HP Reveal was used for creating AR prototypes. Subsequently, the development platforms Unity Vuforia, AR Kit, and MAKAR were used due to their comprehensive features and functionalities for AR development.	School	Communication skills	Quantitative

^a^Participant details are reported here as they appear in the studies

^b^The paper lacks information regarding the age range of the participants. Only the minimum age, age mean, and standard deviation are provided

### RQ1: Characteristics of Projects that Include Autistic People in Extended Reality Technology-based Research

As outlined in the introductory section, the use of assistive extended reality technology has gained popularity in recent years for its potential to enable outcomes for autistic individuals. Given the dearth of literature on the subject the aim of this research question was to explore the characteristics of these projects, including sample size, participant demographics, study design, and outcome measures.

#### Participant Demographics

Autistic demographics are commonly reported alongside characterizations of autism symptom severity, with research studies using terms such as mild, moderate, severe, and profound (Frith & Happé 1994). In addition, terms such as high- and low-functioning are prevalent (Boucher, et al., 2008). These terms have been the subject of some debate (Keating et al., 2023). However, this debate is beyond the scope of the current systematic review. While we acknowledge and recognize this debate, we report the terms exactly as authors use them in the included studies to maintain methodological transparency. Of the 20 studies, 16 provided details regarding the number of autistic people who participated in their studies. The aggregate number of autistic participants across these 17 studies was 314 without considering stakeholders. The study that encompassed the greatest number of autistic participants consisted of 176 people, whereas the study with the fewest participants included only one individual. In regards to the age of the participants, the studies varied from 2 to 22+years old. Five studies exclusively targeted autistic adults (22+years old), five involved young autistic participants (13–21 years old), and six targeted autistic children aged 2 to 12. The majority of participants had a documented diagnosis of “mild” to “moderate” autism (12 articles). Additionally, two (2) studies focused on “low-functioning” autistic participants, and six (6) studies referred to their participants as autistic without further details.

#### Publication Year, Country, and Targeted Outcomes

In further outlining the characteristics of the studies, we next report on the publication year, country and outcomes. Extracting these data from the included research papers provides important contextual information and establishes the relevance of the work included. The range of publication years for the included articles spanned 2002 to 2022 (as per the search criteria), with the preponderance of articles ​​being published in 2019 and 2022 (with five articles each), followed by 2021, with four articles. Out of the 20 articles, six were published in the United States, and four originated from the United Kingdom. Additionally, Taiwan contributed two articles, while Ireland, Canada, Italy, Malaysia, Indonesia, Algeria, China, and Qatar each published one article. In relation to the outcomes measured by the included studies, social and communication skills were the primary targets of the research, with seven studies focusing on enhancing social skills and four studies targeting communication skills. Attention management skills were the focus of three studies, while two studies each targeted emotional and adaptive skills. Further, job interview skills were the focus of two studies, and one study was developed to support inclusive practices in museums.

#### Study Designs and Sample Sizes

The included studies used diverse methodologies and different types of study designs. Of the 20 papers selected, eight were qualitative, four articles used mixed methods, five were quantitative and the three remaining used mixed or multi-methods research (a combination of quantitative and qualitative data sources and analysis techniques). Across studies, reported primary goals differed significantly. Two-thirds of the studies focused on evaluating the usability of their products and the effectiveness of these. Five studies assessed the feasibility and usability of their systems, and the remaining two were a validation study and an adaptation of an evidence-base, respectively. The studies’ sample sizes for autistic participants varied considerably, ranging from one to 176 individuals. Nearly half of the reported literature included fewer than six participants. Additionally, two studies had a sample of fewer than ten autistic adults. On the other hand, four studies had a sample size greater than 18 participants. Meanwhile, four studies did not disclose data regarding their sample sizes. The location where the studies took place varied from face-to-face contexts (e.g., classrooms, learning centers, labs, museums) to virtual (e.g., home), with the majority of included studies conducted in school classrooms (n = 8).

#### Immersive Technology Systems Characteristics

The immersive technologies used in the included studies were also diverse, with six studies using desktop-based VR, four using mobile AR, and four using head-mounted displays. Three studies utilized virtual worlds, and one study used large-scale spatial augmented reality systems. One study employed both AR and VR, while the remaining study used a combination of mobile AR, desktop VR, and Cave automatic virtual environment (CAVE) systems. For instance, VR-JIT, a single-user computerized interview simulator, used a desktop-based Virtual Reality (VR) system operated with a mouse and a joystick (Smith et al., 2020). The AS Interactive project offered both single-user virtual environments and collaborative virtual environments (Cobb et al., 2002). Another project used a desktop-based VR system with a mouse and keyboard and explored the use of collaborative virtual environments in a shared problem-solving activity called Block Party (Millen et al., 2011).

Also, a spherical camera and a smartphone equipped with Android 6.0 OS were used in combination with Ricoh Theta S apps, Ricoh Theta Converter Pro, Retouch3, and Marzipano Tool4 to create and organize a sequence of spherical photos for a virtual tour (Giaconi et al., 2021). Virtuoso includes two applications: Virtuoso-VR, a multi-user virtual environment developed first in High Fidelity and later in Unity, and Virtuoso-SVVR, a single-user 360-degree video mobile application developed in Unity (Schmidt & Glaser, 2021a, b). Some projects used VR in conjunction with other hardware, such as HMDs, Oculus Rift, Kinect devices, and AR markers (Adiani et al., 2022). Finally, in some studies, AR systems were developed for use on mobile phones, with the scenes displayed on a 20-inch monitor (Wang et al., 2022).

### RQ2: What are Reported Outcomes, Recommendations, and Implications of Co-design with Autistic People for Research and Practice?

Research question 2 sought to elucidate the reported outcomes, recommendations, and implications of co-designing research and practice with autistic individuals. Through analysis of the 20 studies included in this review, findings suggest that involving autistic individuals and stakeholders in the research process has several positive outcomes. Firstly, it helps the research outcomes (whatever they may be) to cater to the actual needs of the end-users and makes the experience more acceptable and accessible for autistic people. Eleven studies (Adams et al., 2022; Adiani et al., 2022; Giaconi et al., 2021; Millen et al., 2011; Politis et al., 2019; Ramachandiran et al., 2015; Rutten et al., 2003; Schmidt & Glaser, 2021a, b; Smith et al., 2020) reported that involving autistic individuals in research allowed them to voice their opinions and become more self-aware, which in turn, led to them feeling empowered.

For instance, Smith et al. (2020) found that collaboration with autistic individuals and stakeholders increased the accessibility, acceptability, and transparency of their adapted VR job interview training for transitioning autistic youth. Additionally, eleven of the selected studies reported that incorporating the views of autistic communities in their research allowed them to meet specific requirements of this population, such as making prototypes user-friendly, engaging and enjoyable (Adiani et al., 2022; Cobb et al., 2002; Escobedo et al., 2014; Ip et al., 2022; Giaconi et al., 2021; Ghanouni et al., 2019; Halabi et al., 2017; Millen et al., 2011; Rutten et al., 2003; Schmidt & Glaser, 2021b, Wang et al., 2022) reported that incorporating the views of autistic communities in their research allowed them to meet specific requirements of this population, such as making prototypes user-friendly, engaging and enjoyable. For example, Escobedo et al. (2014) reported positive feedback from participants, who found the product useful, exciting, and easy to use. Similarly, Schmidt and Glaser (2021b) reported that their participants found their prototypes easy to use and encountered fewer usability problems and technology-induced errors. While the majority of the selected studies (19 articles) demonstrated that involving autistic individuals and stakeholders in the research process led to positive outcomes for this population, two studies did not report the outcomes of involving autistic communities in their research.

In terms of the outcomes of participatory design approaches for autistic individuals, six out of the 20 included articles (Adams et al., 2022; Cobb et al., 2002; Giaconi et al., 2021; Halabi et al., 2017; Millen et al., 2011; Rutten et al., 2003) indicated that such involvement promoted a sense of enjoyment, belonging, ownership, and self-efficacy. Additionally, four articles (Cobb et al., 2002; Millen et al., 2011; Politis et al., 2019) demonstrated that involving autistic individuals facilitated the expression of their opinions and perspectives, a skill that can be challenging for this population. Moreover, one study (Adams et al., 2022) observed that by collaborating with autistic communities, researchers were able to decrease anxiety in autistic participants by ensuring appropriate levels of cognitive load in their activities. Finally, another study presentation(Giaconi et al., 2021) underscored that co-designing research with autistic individuals protected their rights by prioritizing their self-determination and self-representation dimensions.

### RQ3: What is the Nature of Inclusion of Autistic People in the Corpus of Included Articles?

The value of involving the autistic communities in the design process has been increasing/has increased (Roche et al., 2021). Thus, this research question seeks to answer: (1) who the research informants were, (2) at what stage of the research autistic people were involved, and (3) how their participation informed the design process.

#### Research Participants

Of the 20 studies included in this review, three studies (Millen et al., 2011; Politis et al., 2019) involved only autistic people as co-designers and participants in their research, while eleven studies (Adiani et al., 2022; Amara et al., 2022; Cobb et al., 2002; Giaconi et al., 2021; Ghanouni et al. 2019; Lee, 2019; Politis et al., 2019; Rutten et al., 2003; Schmidt & Glaser, 2021a, b; Smith et al., 2020; Wang et al., 2022) involved multi-participant groups in the design of their studies, including autistic individuals, parents, teachers, clinicians, therapists, and other stakeholders. The remaining seven studies solely relied on input from stakeholders (e.g. parents, teachers) to design their studies.

#### Involvement of Autistic People and Stakeholders in the Research Process

Our analysis of 20 studies revealed that autistic individuals were engaged at varying levels throughout the research process. Specifically, seven studies (Adiani et al., 2022; Giaconi et al., 2021; Ghanouni et al. 2019; Politis et al., 2019; Ramachandiran et al., 2015; Wang et al., 2022) involved autistic individuals and stakeholders in all phases of the research, from planning to prototype testing to the final stage of the project. Seven studies (Amara et al., 2022; Escobedo et al., 2014; Lee, 2019; Halabi et al., 2017; Ip et al., 2022; Schmidt & Glaser; 2021a, b) solely involved autistic individuals in the testing phase of their product, meaning that these studies relied solely on the information provided by stakeholders and experts to build their prototypes. Furthermore, three studies (Cobb et al., 2002; Millen et al., 2011; Rutten et al., 2003) involved autistic individuals and stakeholders in designing, reviewing, and evaluating their products, allowing for a more collaborative approach to product development. Two studies (Adams et al., 2022; Smith et al., 2020) involved autistic individuals during the planning and problem identification phase of their project. Finally, one study (Miningrum et al., 2021) failed to include autistic individuals in the study's design; however, it did involve therapists and teachers who work with autistic students.

#### How Do Autistic Individuals and Stakeholders Inform the Design Process?

Drawing on the synthesis of the 20 included articles, findings indicate that autistic individuals and stakeholders have played a significant role in shaping those studies’ design processes. Specifically, nine of the reviewed studies (Adams et al., 2022; Adiani et al., 2022; Escobedo et al., 2014; Giaconi et al., 2021; Miningrum et al., 2021; Politis et al., 2019; Schmidt & Glaser, 2021a, b; Wang et al., 2022) reported that involving autistic individuals in the observation of prototypes facilitated the refinement of designs based on the preferences and needs of this population. Six studies (Cobb et al., 2002; Ghanouni et al., 2019b; Millen et al., 2011; Politis et al., 2019; Ramachandiran et al., 2015; Rutten et al., 2003) highlighted that participants were actively involved in the design process as co-designers and co-creators, rather than passive consumers of their final products. For example, Millen et al. (2011) encouraged their participants to become co-designers, testers, and evaluators of prototypes to ensure that the final product was aligned with their preferences and feedback. Finally, five studies (Amara et al., 2022; Halabi et al., 2017; Ip et al., 2022; Lee, 2019; Smith et al., 2020) incorporated feedback from target users, experts, and practitioners to ensure that the final products were appropriate and developed in accordance with feedback from relevant stakeholders. Table 2 details who participants were, when they were involved, and how this supported the design process of the XR technology.

**Table 2:** Information extracted in relation to question 3

Project title authors	Which groups were involved?	Involvement phase	How was design informed?
VR-JIT; Smith et al. (2020)	Autistic people, parents, teachers, community employers	-Problem identification	Autistic adults evaluated the design of VR-JIT by identifying its helpful features and potential barriers, as well as by providing recommendations for improvement. A scientific advisory board then reviewed the recommendations and retained those that were feasible and within the project's scope.
AS interactive; Cobb et al. (2002), Rutten et al. (2003)	Adults with Asperger’s syndrome, teachers, support workers.	-Design -Evaluation	Adults with Asperger’s syndrome participated in reviewing the design and usability of a virtual environment for social skills training. In addition, training professionals served as facilitators to help participants in using the system and to provide feedback to the design team. Finally, teachers were invited to participate in workshops to review content and provide feedback, which was used to refine the design and to develop additional training. The research team engaged in discussion, consultation, and research with teachers, facilitators, and end-users to understand how VEs could be used to support social skills learning and identify important design features. Teaching approaches suitable for autistic individuals were used to gather their feedback and concerns about single-user VEs and collaborative VEs.
COSPATIAL; Millen et al. (2011)	Autistic people	-Design -Evaluation	Autistic participants were encouraged to become critical co-designers, testers, and evaluators of the prototypes. In turn, the final prototypes were created according to their preferences and based on their feedback.
Museum accessibility; Giaconi et al. (2021)	Autistic people, museum didactics experts, special pedagogy experts, AR expert	-Problem identification -Development -Evaluation	The research team collected data about the perceptions of high-functioning autistic people when visiting a museum and analyzed the data to identify necessary accommodations. The research team also visited the museum several times with high-functioning autistic students to collect data through observation. All the information gathered from autistic students was used to inform the construction of a virtual prototype.
VR based behavioural learning; Ramachandiran et al. (2015)	Parents of autistic children	-Problem identification -Development -Evaluation	Parents of autistic children were interviewed to gather information on their children's essential needs and define design criteria for the virtual environment. The parents were also involved in selecting objects related to the environment, and the prototype was designed based on their feedback and choices.
Virtuoso; Schmidt & Glaser (2021a, b)	Autistic people, Autism care providers	-Evaluation	The visual and interactive features of Virtuoso were developed through ongoing conversations with autism care providers and autistic people. Experts with expertise in autism research and prior clinical interactions with autistic adults were also consulted to ensure the design took into account the unique needs of autistic users. Autistic adults took part in two different sessions of approximately 30 min each, and after each session, they completed the system usability scale and one user-friendliness question. Additionally, unstructured interviews were conducted with each participant to learn about their experiences, exploring what they liked best, least, and what they might change.
VL4ASD; Politis et al. (2019), Politis et al. (2020)	Autistic people	-Planning -Design -Evaluation	Autistic individuals actively participated in designing this conversation skills training, leading to the early identification and resolution of issues in the development stages. Feedback sessions with autistic individuals and practitioners ensured that the training material was appropriate and useful for the target audience. A brief questionnaire was used to identify design features that were not sufficiently intuitive and to gather data about reported bugs and users' confidence levels in using the VW. Stress testing was carried out to detect bugs and scrutinize issues. Feedback from autistic people was analyzed and used to improve or add features. This participatory design approach had a positive impact on the final outcome.
MOBIS AR; Escobedo et al. (2014)	Teachers of autistic children, autistic people	-Evaluation	The researchers conducted two participatory design sessions with teachers to discuss prototypes and gather design insights, and based on the results, selected one prototype to redesign and deploy. Following the deployment of the system, the researchers interviewed teachers as proxies for student needs and reactions, as only three of the 12 students could pronounce some words.
AR emotion; Miningrum et al. (2021)	Teachers of autistic people, autism care providers	-Problem identification -Evaluation	The design was improved through the feedback gathered from therapists. Additionally, interviews with teachers from a special school provided data that was used as a reference for designing the story and creating the adapted book illustration.
DIVRS for communication; Halabi et al. (2017)	Autistic people experts and practitioners	-Evaluation	The “greeting” scenario was developed in consultation with experts and practitioners in the local center of education for the autistic, who handle autistic children on a daily basis. An exit interview was conducted with autistic participants at the end of the experiment to acquire feedback about the satisfaction and impact of immersion level on them.
ARCB; Lee, I. J. (2019)	Parents of autistic children, teachers of autistic people	-Evaluation	The creation of social scenario stories was based on the interviews conducted with participants' teachers and parents and a survey of daily situations according to their social network.
VR social stories; Ghanouni et al. (2019)	Autistic people, parents clinicians	-Planning -Design -Evaluation	A committee of stakeholders consisting of a high-functioning autistic youth, parents of autistic children, and clinicians provided insight into setting up the structure of the study and helped develop initial scenarios. Two main sessions with two iterations each were conducted to obtain consensus on the content of socio-emotional scenarios for a gaming program and to validate the type and intensity of emotions for each scenario. Feedback and suggestions from stakeholders were used to revise the content, emotion type, or intensity level for scenarios that did not reach an agreement, resulting in multiple iterations of revisions.
VRCBT for social anxiety; Adams et al. (2022)	Autistic people, parents of autistic children, autism care providers	-Planning	Clinicians were consulted first before finalizing the agenda for the advisory meeting with the young people and parents. Consensus was reached across advisory meetings. Feedback gathered from end users or stakeholders and was used to improve the VR environment developed
CIRVR; Adiani et al. (2022)	Autistic people, job coaches, career counselors, employers, support professionals	-Planning -Development -Evaluation	The VR interviewing system was modified based on input from autistic individuals and stakeholders. Changes were implemented during development after testing with 4 autistic self-advocate interns, and feedback from 9 autistic participants during the testing phase also led to further improvements.
VR for affective expression and social reciprocity; Ip et al. (2022)	Teachers of autistic people	-Evaluation	Teachers were in constant communication with the research team to help identify the potential improvements that could be made to both the VR learning scenario designs and the overall design of the training programme. Schoolteachers were invited to collaborate with the research team in co-designing the social contexts and situations in the school environment.
AR hand gesture and voice interaction; Amara et al. (2022)	Teachers of autistic people, autism care providers	-Evaluation	All design development was discussed initially with educators and therapists.
Requesting help AR; Wang et al. (2022)	Parents of autistic children, teachers of autistic people, autism care providers	-Planning -Development -Evaluation	In stage one, core vocabulary graphics were developed through regular meetings between parents, class tutors, and language therapists, which were then used to create an interface for participants to practice specific sentence patterns. In stage three, the prototype was tested by parents and caregivers to evaluate its usability for autistic children. Finally, the effectiveness of the refined interface was assessed by designers, special education instructors, and speech therapists.

## Discussion

This systematic review reveals that information is limited in the field of XR reporting on the involvement of autistic people through co-design and participatory frameworks. In total, across 20 years of research, we located 20 articles within the scope of our search criteria that involved autistic groups in participatory and co-designed research. Almost half of these studies (n = 10) were located in either the USA (n = 6) or the UK (n = 4). Of these, five of the studies were performed by just two research teams. This suggests a narrowing of diversity in relation to scholars undertaking this research. However, analysis highlights that work in this area is increasing, but only in a limited way. Specifically, research has increased from one article a year in 2002 to five per year in 2019, after which publication rates remain between four and five articles a year. This growth could signal that researchers are taking heed of increasing calls for inclusion of autistic people in XR research (Gillespie-Lynch et al., 2021; Nicolaidis et al., 2019). However, uptake of participatory and co-designed research remains limited, which is concerning, given that “the last decade has witnessed the emergence of a powerful call from autistic people to have real input into the decisions that shape their lives” (Poulsen et al., 2022, p. 3).

### Discussion of Findings Related to Research Question 1

Research question 1 was concerned with the characteristics of published projects that include autistic people in extended reality technology-based research. A range of noteworthy themes emerged regarding the characteristics of these projects. First, desktop-based virtual reality XR systems were the most prominent, especially in early research efforts, while head-mounted displays and platforms built upon software such as Unity facilitated greater immersion and opportunities for interactions in real-time. Mobile augmented reality systems used everyday devices (i.e., smartphones) to bridge digital content with physical objects, providing unique opportunities for therapy and education.

Moreover, several studies focused on developing software content or protocols for future use in XR, indicating ongoing innovation in this field. In exploring this research question, we found disparities regarding technology semantics. For example, Smith et al. (2020) described a multimedia, video-based project as VR; however, definitions of VR (Hale & Stanney, 2014) would not align to this particular technological setup. Alternatively, Schmidt & Glaser (2021a) also designated a multimedia video-based project as VR, but they employed 360-degree videos and VR headsets, effectively placing this project within an established VR classification. Further, Amara et al. (2022) described some of their VR activities as being ‘game-like’, further muddying the waters in defining these immersive experiences. Such disparities are acknowledged by Savickaite and colleagues (2022), who suggest that: “[...] in the absence of transparent reporting standards and terminology, readers may be left confused (or unintentionally misled) by manuscripts that use the term ‘virtual reality’ to describe non-HMD devices” (np). A final point related to this research question is the nature of anticipated outcomes and research foci of the studies included. We found the earlier work focused mostly on social skills (Cobb et al., 2002; Rutten et al., 2003; Millen et al., 2011) while the later work presented more diverse and wide-ranging foci. This included (but not limited to): Promoting cultural access (Giaconi et al., 2021); public transportation training (Schmidt & Glaser, 2021a, b); and job interviewing skills (Adiani et al., 2022; Smith et al., 2020). This diverse work was published between 2020 and 2022 and suggests that research might be evolving towards research agendas and foci that are more in alignment with the needs of the autistic community; more than they have been in the past.

Most autism research with XR has historically been conducted with children, often without significant contribution from autistic adults (Happé & Frith, 2020). However, the findings of this study indicate a shift in this pattern, with an increasing number of studies involving older individuals. Despite this progress, there is still a substantial need for greater involvement of autistic adults in research. Furthermore, researchers will need to consider meaningful ways to engage younger groups in co-design to ensure their voices are also included (Newbutt et al., 2023).

### Discussion of Findings Related to Research Question 2

Research question 2 sought to identify reported outcomes, recommendations for, and implications of participatory and co-design with autistic people for research and practice. According to Parsons and Cobb (2013) and Fletcher-Watson et al. (2019), considering the views of autistic individuals and stakeholders throughout the design process can improve the quality of research methods and lead to better translation of research into practice, and improved outcomes for autistic people. Other research and reviews came to the same conclusion (Tsikinas & Xibigalos, 2019). Therefore, it is especially interesting to note that our review located only 20 articles that report on, or included, autistic input to XR-based research. Moreover, XR-based research and technologies are especially well-placed to champion and utilize their design to include user-centered design processes (Bauer et al., 2022). This systematic literature review identified a body of experimental and applied research that highlights the benefits of involving the autistic community in priority-setting in XR-based research. Consequently, several recommendations for future research have emerged from the papers reviewed. Smith et al. (2020) outlined that careful attention should be given to cognitive load and gamification techniques when designing with this heterogeneous population. In line with this suggestion, Giaconi et al. (2021) recommended that scholars develop integrated systems of technologies capable of creating museums accessible to neurodiverse people. Moreover, the previous research of Rutten et al (2003) supports the argument for integrated systems in the newer Giaconi research, which cautioned researchers that VR alone may not be adequate as a training tool for autistic individuals, and additional support may always be necessary. Additionally, two studies (Politis et al., 2019; Schmidt & Glaser, 2021a) called for more longitudinal studies to collect research data and answer questions concerning the novelty effect of extended reality technologies because it is difficult to keep abreast of the rapid pace of technological evolution. Finally, Howard and Gutworth (2020) have suggested that future research include a wider population with larger sample size, and design immersive spaces to be more reflective of real life and tailor their training to meet autistic individual needs and preferences. We also found that two studies did not report the outcomes of involving autistic communities in their research; and we suggest that moving forward in this field, this is something researchers not only consider but include as part of their approach to including and engaging autistic people in research.

In spite of the benefits of involving autistic individuals in the design process, there are some challenges associated with this approach. Four out of the 20 selected articles highlighted these challenges (Cobb et al., 2002; Politis et al., 2019). First, stakeholders may have divergent goals and perspectives, which researchers must be able to integrate into a cohesive design process (Cobb et al., 2002; Politis et al., 2019). Second, some autistic individuals may struggle to articulate their opinions and require tailored or alternative approaches to elicit their perspectives (Cobb et al., 2002; Politis et al., 2019; Millen et al., 2011). Third, accessibility issues and barriers presented by researchers may impede the meaningful engagement of some autistic individuals in the design process (Millen et al., 2011). Fourth, co-designing with autistic communities requires significant time and effort (Millen et al., 2011). Finally, accommodating all recommendations from this diverse population may not align with the ethical guidelines of Institutional Review Boards (IRBs) (Politis et al., 2019) due to the complex nature of this process that does not always require researchers to think in ways to engage and involve diverse populations in their research fully. There can also be a lack of understanding or empathy from neurotypical researchers, which can lead to miscommunications or misguided assumptions about the needs and abilities of autistic individuals

### Discussion of Findings Related to Research Question 3

Research question 3 investigated the nature of inclusion of autistic people in the corpus of included articles. Findings suggest that autistic people actively participated in various stages, including design, evaluation, and problem identification. Further, parents of autistic children and teachers of autistic individuals also were actively involved in problem identification, design, and evaluation stages. In addition, and in some cases, autism care providers, clinicians, and experts in autism were consulted to ensure the design considered the unique needs of autistic users. These groups were involved in various inclusive design practices, including participatory design, co-design, testing, and evaluation, leading to the identification and resolution of issues and enhancement of designs through solicitation of preferences, feedback, and recommendations to inform research and development. While each of the studies we reviewed included various levels and modalities of autistic input on the research and development process, none of the studies reported including autistic people or their stakeholders in co-creation of research questions or design methodologies. This suggests that research agendas may not be driven by autistic preferences, but instead by researchers who are presumably not autistic; thus, it would seem that a research gap exists relative to guiding research agendas within XR-based research for autism (Poulsen et al., 2022).

**Fig. 2:**
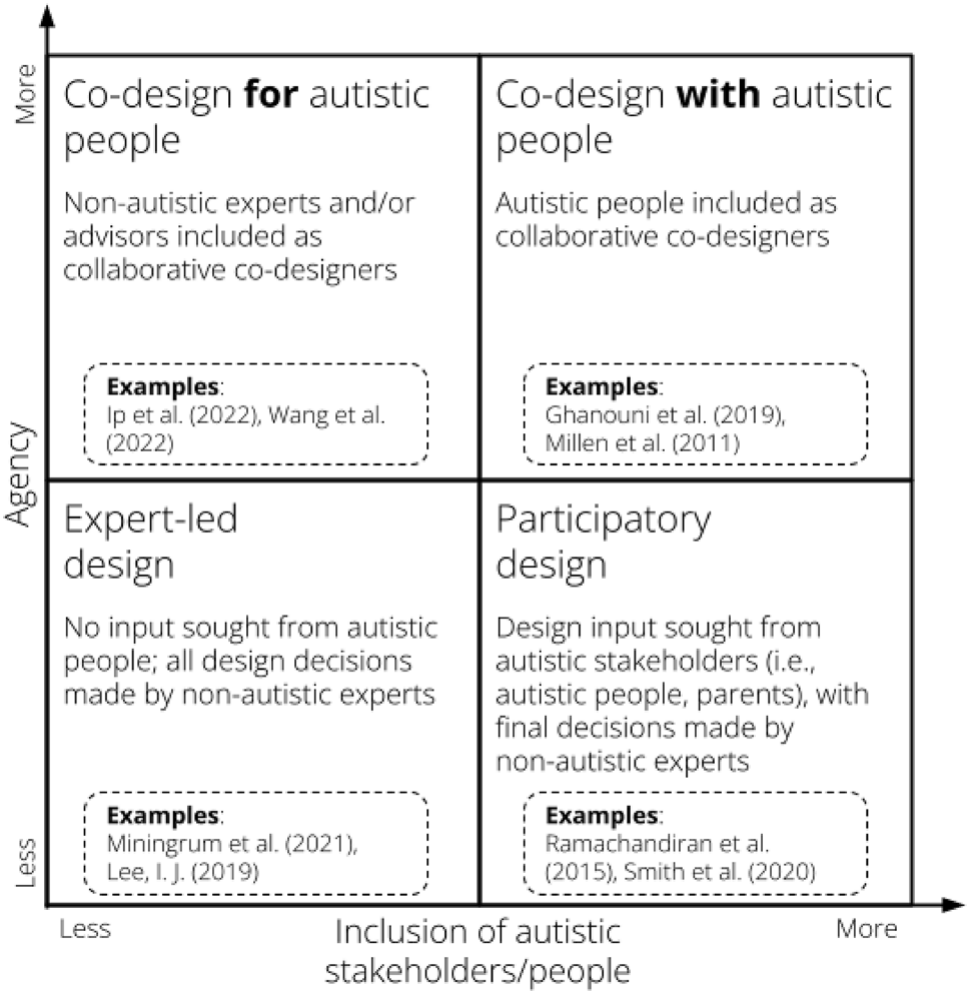
Dimensions of inclusion in design of XR with autistic populations framework including illustrative examples from the systematic review

Based on insights gained from findings reported in Table 1 on inclusive design practices across various projects and stakeholders, including autistic individuals, parents, teachers, clinicians, and experts, we propose a design framework that characterizes autistic inclusion in XR research (Fig. 2).

The framework consists of four categories that describe autistic inclusion in XR research and development. These include: (1) expert-led design, (2) participatory design, (3) co-design for autistic people, and (4) co-design with autistic people. These categories are illustrated in Fig. 2 within four quadrants located on X and Y axes. The X axis represents agency, that is, the ability to influence design decisions related to the XR application.. The Y axis represents inclusion of autistic people and stakeholders, that is, the degree to which autistic people are included in XR research and development processes. The X and Y axes represent increasing agency and inclusion respectively.

The top two quadrants of Fig. 2 encompass those studies that utilized a co-design approach. By co-design, we refer to those design approaches that, in contrast with top-down approaches to design in which a user’s role is more passive, users are active participants in the design process who are valued as equal contributors (Roschelle et al., 2006). The bottom two quadrants represent those studies that used a participatory design approach. By participatory design, we refer to design processes that bear some similarity to co-design, but differentiate themselves in that users are not seen as equal contributors. That is, user input is solicited in participatory design, but the design team is ultimately the arbiter of decision-making (Engelbertink et al., 2020).

Within the top two quadrants of Fig. 2, we differentiate co-design as being either *for* autistic users (top-left quadrant) or *with* autistic users (top-right quadrant). Within the included studies, co-design *for* autistic users involved non-autistic participants, such as experts or parents, who take an active role alongside the research team in the design process. Co-design *with* autistic users was illustrated by research teams actively involving autistic users as designers, with design input and decision-making capabilities equal to those of the research team. Within the bottom two quadrants of Fig. 2, we differentiate participatory design as being either led by non-autistic experts (bottom-left quadrant) or being conducted with autistic people/stakeholders (bottom-right quadrant). In the included corpus of articles, non-autistic experts included teachers, therapists and medical professionals who were often included in providing input and feedback on XR designs. In contrast to this, autistic stakeholders included parents, caregivers, providers, and teachers who were invited to provide input and feedback based on their direct involvement with autistic people (i.e. as their children or students) or lived experiences as an autistic person. Within the participatory design frame, neither non-autistic experts nor autistic stakeholders had agency to make final design decisions. We illustrate these quadrants with examples in the following paragraphs.

### Expert-led Design

Two articles using the expert-led design approach were Miningrum et al. (2021) and Lee. (2019), in which the design decisions are primarily made by non-autistic experts without seeking direct input from autistic individuals. Miningrum et al. (2021) describes how the design process involved input from teachers of autistic people and autism care providers, with design improvements based on feedback gathered from therapists. Similarly, Lee (2019) reports how parents and teachers of autistic children were interviewed to inform creation of social stories; however, there is no mention of involving autistic individuals themselves in the design process. Both articles demonstrate an expert-led design approach, in which design decisions were made by non-autistic experts, such as teachers, parents, and therapists, without seeking direct input from autistic individuals. The absence of direct input from these individuals indicates that the design was primarily driven by the knowledge and understanding of the non-autistic professionals involved.

### Participatory Design

Examples of participatory design approaches were found in Ramachandiran et al. (2015) and Smith et al. (2020), in which design input was sought from autistic stakeholders (such as autistic individuals themselves and their parents), but final design decisions were still made by non-autistic experts. Ramachandiran et al. (2015) report a design process that involved active participation from parents of autistic children who were interviewed to gather information about their children's essential needs. In this case, the input of autistic stakeholders (in this case, parents) played a significant role in shaping the design decisions, but these design decisions were not made by stakeholders. Similarly, Smith et al. (2020) report a design process that involved input from multiple autistic stakeholders, including autistic people, parents, teachers, and community employers, with stakeholders providing recommendations for improvement. However, a non-autistic scientific advisory board reviewed the recommendations and made final design decisions. Both articles outline participatory design approaches where input from autistic stakeholders, such as autistic individuals and parents, was actively sought to inform the design process. However, the final decisions were made by non-autistic experts, which raises questions regarding the balance between incorporating the input of autistic stakeholders and considering the expertise and knowledge of non-autistic professionals.

### Co-design for Autistic People

Two examples of co-design for autistic people using a co-design approach were Ip et al. (2022) and Wang et al. (2022), in which the design process involved collaborative input from both non-autistic experts and representatives for autistic people. Ip et al. (2022), included teachers of autistic people who played a vital role in the evaluation phase. Likewise, Wang et al. (2022), included autistic stakeholders in the planning, development, and evaluation stages, showcasing a co-design process involving multiple stakeholders (parents of autistic children, teachers of autistic people, and autism care providers). Both articles demonstrate a co-design process where non-autistic experts, such as parents, teachers, researchers, and therapists, actively collaborated to create XR experiences for autistic people.

### Co-design with Autistic People

Two studies shared examples of co-design where autistic people were actively involved in the design process and incorporated their perspectives and preferences (Ghanouni et al., 2019; Millen et al. 2011). Data were collected regarding their perceptions and experiences when visiting a museum, and were analyzed to identify necessary accommodations. The research team further collaborated with autistic students by observing their museum visits, thus allowing for direct input from the autistic community. These data, combined with observations, informed the creation of a virtual prototype that aimed to address the identified needs and preferences of autistic individuals. Similarly, Millen et al. (2011) involved autistic participants as critical co-designers, testers, and evaluators in the development of prototypes. By actively engaging autistic individuals in the design process, their unique insights and feedback were incorporated into the creation of the final prototypes. The preferences, perspectives, and experiences of autistic participants were given significant importance, ensuring that the end products were tailored to meet their specific requirements. By actively involving autistic individuals in the design process, both studies valued and prioritized autistic perspectives.

### Limitations

The findings reported here should be viewed in the context of the following limitations. Firstly, we did not include so-called “gray” literature to ensure included studies were of peer-review quality. In doing so, our review neglected a range of outlets (e.g., conference proceedings, published dissertations) which might have yielded work reporting co-design approaches used in research with autistic people. In addition, book chapters were also discounted that might have provided insights of research reporting co-design and participatory-based research within XR domains and fields. Another limitation relates to the terminology used in our search terms, such as neurodiverse/neurodiversity, cognitive disabilities, developmental disabilities, and other related terms, such as user-experience (UX), social robots, or AI, which could have led to missing studies that conceive of or describe co-design and in ways that may be somewhat divergent. In addition, we only searched for articles in English, thereby potentially introducing biases as the work presented would likely exclude research from non-White, the Global South, and intersectional researchers and autistic individuals. Inclusion of non-English articles may have increased the number included in our corpus.

## Conclusion

With increased calls from the autistic community to include their perspectives in research that are intended to inform subsequent practices and support them, this paper presents a systematic literature review aiming at identifying the degree to which autistic individuals have been involved in co-designing extended reality research over a twenty-year period spanning from 2002 to 2022. The review analyzed 836 articles from which 20 publications were selected for assessment. The collected evidence indicates that there is a growing trend to involve the autistic community in research projects that focus on XR technologies, particularly in the last three years of this review. This trend could be due to researchers making a conscious effort to solicit user input, rely on stakeholders as proxies for design input, and involve users directly in the design and the testing phases of their research. However, there remains a significant gap that must be addressed, given that most XR research related to autism is still conducted on autistic people rather than in collaboration with them. Additionally, the findings from this systematic literature review suggest that involving the autistic community as co-designers in all stages of research is an effective strategy for identifying the desired outcomes and preferences of the community, ultimately resulting in more relevant and impactful outcomes. Nonetheless, there are challenges associated with co-designing with this population due to their neurodiversity; sometimes necessitating the use of custom, tailored, or substitute methods to gather their viewpoints. Finally, given the participation of various stakeholders, including parents, siblings, special education teachers, school staff, healthcare professionals, and autism advocates in the studies selected, it is crucial to ascertain the extent of autistic involvement in the research process to ensure that the research outcomes are reflective of the needs of this population. Also, based on the diversity of research participants, it is currently unknown whether the perspectives and preferences of autistic individuals differ from those of stakeholders. Thus, future research endeavors could benefit from focusing on this aspect to gain a comprehensive understanding of the priorities of different groups that factor in race, gender, and intersectionality.

## Supplementary Information

Below is the link to the electronic supplementary material.

Supplementary material 1 (DOCX 18.0 kb)

## Footnotes

1 We adopt language in our research that is preferred by the autistic community. In addition, we recognize, as Bottema-Beutel and colleagues (2021) do, that “language is a powerful means for shaping how people view autism” (P.19). Therefore, we use terms in our work accordingly and in line with the autistic communities preferences (Kenny et al., 2016).
