# Supplementary material for: How are Autistic People Involved in the Design of Extended Reality Technologies? A Systematic Literature Review
Source: J Autism Dev Disord. 2023 Sep 16;54(11):4232–58. doi: 10.1007/s10803-023-06130-3 (PMC11461589; doi:10.1007/s10803-023-06130-3)
Supplement: Supplementary file 1 — Supplementary material 1 (DOCX 18.0 kb) [file 10803_2023_6130_MOESM1_ESM.docx]

| **Appendix A**. Database and Search Terms | |
| --- | --- |
| Database Search Query | |
| Scopus | ( TITLE-ABS-KEY ( autis* OR asd OR asc OR asperg* ) AND TITLE-ABS-KEY ( ( virtual AND reality ) OR ( extended AND reality ) OR ( augmented AND reality ) OR ( mixed AND reality ) OR ivr OR ( virtual AND worlds ) OR ( virtual AND environment ) OR ( cross-reality ) OR immers* ) AND TITLE-ABS-KEY ( ( end AND user* ) OR ( user AND involvement ) OR ( co-design ) OR ( collaborat* AND design ) OR ( co-development ) OR stakeholders OR participation OR communit* OR ( user-centred AND design ) OR ( user-centered AND design ) OR ucd OR ( desig* AND for ) OR ( caregivers ) OR ( care-givers ) OR ( parent* ) ) ) AND ( LIMIT-TO ( PUBSTAGE , "final" ) ) AND ( LIMIT-TO ( DOCTYPE , "ar" ) ) AND ( LIMIT-TO ( LANGUAGE , "English" ) ) |
| Web of Science | (TI=autis* or TI=ASD or TO=ASC or TI=asperg*) AND (TI=virtual reality OR TI=Extended Reality OR TI=Augmented Reality OR TI=Mixed Reality OR TI= IVR OR TI=virtual worlds OR TI=virtual Environment OR TI=Cross-reality OR TI=Immers*) AND (TI=end user* OR TI=user involvement OR TI=co-design OR TI=collaborat* design OR TI=co-development OR TI=stakeholders OR TI=participation OR TI=communit* OR TI=user-centred design OR TI=user-centered design OR TI=UCD OR TI=desig* for OR TI=caregivers OR TI=care-givers OR TI=parent*) |
| PubMed | ((autis*[Title/Abstract] OR ASD[Title/Abstract] OR ASC[Title/Abstract] OR asperg*[Title/Abstract]) AND ((virtual reality[Title/Abstract]) OR (Extended Reality[Title/Abstract]) OR (Augmented Reality[Title/Abstract]) OR (Mixed Reality[Title/Abstract]) OR IVR[Title/Abstract] OR (virtual worlds[Title/Abstract]) OR (virtual Environment[Title/Abstract]) OR (Cross-reality[Title/Abstract]) OR Immers*[Title/Abstract])) AND ((end user*[Title/Abstract]) OR (user involvement[Title/Abstract]) OR (co-design[Title/Abstract]) OR (collaborat* design[Title/Abstract]) OR (co-development[Title/Abstract]) OR stakeholders[Title/Abstract] OR participation[Title/Abstract] OR communit*[Title/Abstract] OR (user-centred design[Title/Abstract]) OR (user-centered design[Title/Abstract]) OR UCD[Title/Abstract] OR (desig* for[Title/Abstract]) OR (caregivers[Title/Abstract]) OR (care-givers[Title/Abstract]) OR (parent*[Title/Abstract])) Filters: English, from 2006 - 2022. |
| Academic Search Premier | AB ( autis* or ASD or ASC or asperg* ) AND AB ( (virtual reality) or (Extended Reality) or (Augmented Reality) or (Mixed Reality) or IVR OR (virtual worlds) OR (virtual Environment) or (Cross-reality) or Immers* ) AND AB ( (end user*) or (user involvement) or (co-design) or (collaborat* design) or (co-development) or stakeholders or participation or communit* or (user-centred design) or (user-centered design) or UCD or (desig* for) or (caregivers) or (care-givers) or (parent*) ). |

**Appendix B**. Inclusion and exclusion criteria

| Inclusion criteria |
| --- |
| - Peer-reviewed journal articles - Published in English - Investigating the experiences of autistic individuals or stakeholders using immersive technologies that include augmented reality, mixed reality, virtual reality, and 3D virtual worlds. - Report use of participatory, co-design, or other inclusive design approaches - Published from 2002 to 2022 - Articles available in full text - Empirical studies |
|  |
|  |
|  |
|  |
|  |
| Exclusion criteria |
| - Book chapters, proceedings, meeting abstracts, editorials, retracted publications, theses, and reviews. - Studies written in languages other than English - Conceptual or descriptive studies - Investigating the experiences of autistic individuals or stakeholders using immersive technologies that include technologies not within the scope of the review (e.g., games even if displayed within a head-mounted display). - Studies where autistic people are included, but the broader approach is for the full range of any existing developmental or intellectual condition. - Studies do not provide sufficient details of empirical research design and data analysis. - Articles in which the full text was unavailable through interlibrary loan and after contacting author(s). - XR technology used to diagnose instead of providing some kind of intervention, training, or educational experience. |
|  |
|  |
|  |
|  |
|  |

**Appendix C**. Unstructured database search query consisting of search term and Boolean operators

|  | | | | |
| --- | --- | --- | --- | --- |
| Term One | Condition | Term Two | Condition | Term Three |
| Autis* OR ASD OR ASC OR asperg* | AND | (Virtual reality) OR (Extended Reality) OR (Augmented Reality) OR (Mixed Reality) OR IVR OR (virtual worlds) OR (virtual Environment) OR (Cross-reality) OR Immers* | AND | (End user*) OR (user involvement) OR (co-design) OR (collaborat* design) OR (co-development) OR stakeholders OR participat* OR communit* OR (user-centred design) OR (user-centered design) OR UCD OR (desig* for) OR (caregivers) OR (care-givers) OR (parent*) |

**Appendix D.** *Filters or limits applied to each index used in the literature review*

|  | | |
| --- | --- | --- |
| **Databases** | **Search within** | **Filters or Limits Applied** |
| *Web of Science* | Topic | Published between 2002-2022; Article, Review Article, Early Access; English |
| *PubMed* | Title/Abstract | Published between 2008-2022; Full Text; English |
| *Scopus* | Article Title, Abstract, Keywords | Published between 2002-2022; Article; Journal; English |
| *Academic Search Premier* | Abstract or Author Supplied Abstract | Published between 2002-2022; Academic Journals; Peer Reviewed; English |

.
